# Association between severe chronic kidney disease defined by cystatin-c and creatinine and clinical outcomes in an elderly population - an observational study

**DOI:** 10.1590/2175-8239-JBN-2020-0092

**Published:** 2020-11-30

**Authors:** Joana Tavares, Josefina Santos, Filipa Silva, João Oliveira, Jorge Malheiro, Andreia Campos, António Cabrita

**Affiliations:** 1 Centro Hospitalar Universitário do Porto, Largo do Prof. Abel Salazar, Serviço de Nefrologia, Porto, Portugal.

**Keywords:** Renal Insufficiency, Chronic, Creatinina, Cystatin C, Aged, Glomerular Filtration Rate, Outcome Assessment, Health Care, Insuficiência Renal Crônica, Creatinine, Cistatina C, Idosos, Taxa de Filtração Glomerular, Avaliação de Resultados em Cuidados de Saúde

## Abstract

**Introduction:**

Estimated glomerular filtration rate (eGFR) based on serum cystatin-C (sCys) seems as accurate as when based on serum creatinine (sCr), but sCys seems a better predictor of adverse outcomes. We aimed to study whether sCys could be a reliable tool for the prediction of adverse outcomes in elderly patients with severe chronic kidney disease (CKD).

**Methods:**

A group of 348 elderly patients with non-end-stage CKD (stages 1-4, according to eGFR-EPI sCr and/or sCys), referred to our consultation unit during 2016, was retrospectively studied and divided into four exclusive categories: CKD_stage4_neither (eGFR-sCr≥30mL/min; eGFR-sCys≥30mL/min), CKD_stage4_sCr_only (eGFR-sCr<30mL/min), CKD_stage4_sCys_only (eGFR-sCys<30mL/min) and CKD_stage4_combined (eGFRsCr<30mL/min; eGFR-sCys<30mL/min). Baseline characteristics, predictors of death, and clinical events (cardiovascular events and admissions for cardiovascular, acute kidney injury or infectious events) were explored until December 2018.

**Results:**

A 77±7.4 year-old cohort, with a modified Charlson Comorbidty Index (mCCI) of 3 (IQR:1-4), was followed-up during 29 (IQR: 26-33) months. There were no significant differences between the characteristics of the stage 4 groups. Survival analysis was stratified by follow-up at 12 months, and in the first year, survival curves of CKD_stage4_sCys_only and CKD_stage4_combined groups were significantly lower than the other groups (p=0.028). Adjusting for age, sex, and mCCI, CKD_stage4_sCys_only, conversely to CKD_stage4_sCr_only, had higher rates of clinical events (p<0.05) than CKD_stage4_neither group.

**Conclusion:**

In elderly patients with discordant CKD staging, sCys-based eGFR seems to be a better predictor of adverse outcomes than sCr-based eGFR. Patients with stage 4 CKD defined by sCr alone seem to behave similar to those with less severe CKD.

## RESUMO

**Introdução:**

A taxa estimada de filtração glomerular (TFGe) com base na cistatina-C sérica (Cis-C) parece ser tão precisa quanto aquela baseada na creatinina sérica (Cr), mas cis-C parece ser um melhor preditor de resultados adversos. Nosso objetivo foi avaliar se a cis-C poderia ser uma ferramenta confiável para a previsão de desfechos adversos em pacientes idosos com doença renal crônica grave (DRC).

**Métodos:**

Um grupo de 348 pacientes idosos com DRC em estágio não terminal (estágios 1-4, de acordo com TFGe-EPI Cr e/ou Cis-C), encaminhados para nossa unidade de consulta durante 2016, foi estudado retrospectivamente e dividido em quatro categorias exclusivas: DRC_estágio 4 nenhum (TFGe-Cr≥30mL/min; TFGe -Cis-C≥30mL/min), DRC_estágio 4_Cr apenas (TFGe-Cr <30mL/min), DRC_estágio 4 _Cis-C_apenas (TFGe-Cis-C <30 mL/min), DRC_estágio4_combinado (TFGe-Cis-C <30mL/min. TFGe-Cr <30mL/min). Características basais, preditores de óbito e eventos clínicos (eventos cardiovasculares e internações por doenças cardiovasculares, lesão renal aguda ou eventos infecciosos) foram explorados até dezembro de 2018.

**Resultados:**

Uma coorte de 77 ± 7,4 anos, com índice de comorbidade de Charlson modificado (mCCI) de 3 (IQR: 1-4), foi acompanhada durante 29 (IQR: 26-33) meses. Não houve diferenças significativas entre as características dos grupos no estágio 4. A análise de sobrevida foi estratificada pelo acompanhamento aos 12 meses, sendo que no primeiro ano, as curvas de sobrevida dos grupos DRC_estágio4_Cis-C_apenas e DRC_estágio4_ combinado foram significativamente inferiores quando comparadas com os restantes grupos (p = 0,028). Ajustando para idade, sexo e mCCI, DRC_estágio4_Cis-C_apenas, ao contrário do grupo DRC_estágio4_Cr_apenas, teve maiores taxas de eventos clínicos (p <0,05) do que o grupo DRC_estágio4_nenhum.

**Conclusão:**

Em pacientes idosos com estadiamento discordante da DRC, a TFGe baseada na Cis-C parece ser um melhor preditor de resultados adversos do que a TFGe baseada na Cr. Pacientes com DRC em estágio 4, definida apenas por Cr, parecem se comportar de forma semelhante àqueles com DRC menos grave.

## Introduction

Chronic kidney disease (CKD) is not only a risk factor for end-stage renal disease (ESRD), but it is also associated with hospitalizations, cardiovascular disease (CVD), and death 1.

Glomerular filtration rate (GFR) is the standard renal measure and its estimation (eGFR) accuracy is important to detect and stage CKD, as well as to stratify the patients’ risk. It is still unknown if the most accurate GFR estimates correspond to the best clinical risk predictor 2.

Great efforts have been made to determine which is the most suitable method for GFR estimation in the elderly. Flamant et al., in a study comparing Cockcroft-Gault (CG), 4-variable Modification of Diet in Renal Disease (MDRD) Study and CKD Epidemiology Collaboration (CKD-EPI) equations in 786 elderly patients recommended the use of the MDRD Study and CKD-EPI equations rather than the CG equation 3. Plus, when compared to the MDRD equation, the Chronic Kidney Disease Epidemiology Collaboration (CKD-EPI) equations seem to be superior in estimating GFR and in predicting the risk for adverse clinical outcomes, particularly in elderly 4. On the other hand, despite BIS equations having been designed for older adults, little evidence exists showing that these equations improve patient outcomes prediction 5 ^,^ 6.

Several studies have suggested that the use of serum cystatin C (sCys), a marker less susceptible to metabolic and extra-renal factors than serum creatinine (sCr), for eGFR calculation significantly improves the risk classification for death, cardiovascular disease, and ESRD 7 ^-^ 10. This observation illustrates the usefulness of cystatin C in the elderly with CKD, in whom important decisions about CKD management and ESRD preparation have to be considered, as it may allow us to better predict CKD progression and appreciate the competitive ESRD versus death risk 11.

The aim of this study was to evaluate, in a cohort of elderly CKD patients, the association of severe CKD (stage 4) defined by either sCr or sCys alone, or by both, with all-cause mortality and progression to ESRD, and secondly, with cardiovascular events and in-hospital admissions (all-cause, and for infection or acute kidney injury (AKI)). We had hypothesized that CKD stage 4 defined by sCys-based equations could identify patients at a higher risk for worse outcomes and events.

## Subjects and Methods

### Study Population

This longitudinal retrospective study included all patients forwarded to our Nephrology outpatient clinic during the year of 2016 (between the 1^st^ of January and the 31^st^ of December). We studied 348 patients aged over 65 years who had non-ESRD (CKD except stage 5) according to KDIGO 2012 criteria 12.

Our research team, composed of nephrologists only, collected data from electronic medical reports. Besides age and gender, baseline medical history including diabetes, hypertension, dyslipidemia, smoking status, body mass index (BMI), and cardiovascular disease (CVD) was obtained to exclude any potential bias. CVD included coronary artery disease, congestive heart failure, classified by New York Heart Association from stage I to IV, arrhythmia, peripheral artery disease, and cerebrovascular disease. Coronary artery disease was defined as history of myocardial infarction, coronary artery bypass grafting, or coronary stent implantation. Peripheral artery disease was defined as the presence of intermittent claudication or if peripheral revascularization or amputation was performed. Cerebrovascular disease included both previous transient ischemic attacks and stroke. 

A modified version of Charlson Comorbidity Index (mCCI) was calculated. This version excludes patients’ age and CKD status 13 ^,^ 14.

Fasting blood samples were collected at baseline and analyzed in the same laboratory with standardized methods. Serum creatinine was analyzed using a calibrator for automated system (Roche Diagnostics) and serum cystatin C was measured by a particle-enhanced nephelometric assay (DADE-Behring, Siemens Company, European Format)^15^.

eGFR was estimated by the equations derived from the CKD-EPI: CKD-EPI creatinine equation (eGFR-sCr) and CKD-EPI cystatin C equation (eGFR-sCys) 16 ^,^ 17.

Patients were divided into four exclusive categories, meaning that no participant of each group could be part of any other:

**1. CKD stage 4 neither** (eGFR-sCr≥ 30 mL/min/1.73m2; eGFR-sCys ≥30 mL/min/1.73m2) - *the reference group*

**2. CKD stage 4 sCr only** (eGFR-sCr< 30 mL/min/1.73m2; eGFR-sCys ≥30 mL/min/ 1.73m2)

**3. CKD stage 4 sCys only** (eGFR-sCr≥30 mL/min 1.73m2; eGFR-sCys <30 mL/min /1.73m2)

**4. CKD stage 4 combined** (eGFR-sCr < 30 mL/min/1.73 m2; eGFR-sCys < 30 mL/min/1.73 m2

### Assessment of Clinical Outcomes

The primary outcomes of the study were all-cause mortality and ESRD defined as the first day of renal replacement therapy initiation. Death was verified by the electronic death certificate.

Secondary outcomes were cardiovascular (CV) events, defined as events secondary to coronary artery disease, congestive heart failure, transient ischemic attack, stroke, and peripheral artery disease; all-cause hospitalization; admissions due AKI, defined by the 2019 ICD-10-CM diagnosis code N17; and infectious events.

Follow-up was calculated from initial evaluation until death or until December 31, 2018. 

### Statistical Analysis

Baseline characteristics were reported as mean ± standard deviation (SD) and median (inter-quartile range) for continuous variables or as number and percentage for categorical variables. Comparisons of baseline characteristics between stage 4 groups (**stage 4 neither** was excluded) were explored using the Kruskal-Wallis test for continuous variables and chi-square test for categorical variables.

In the following statistical analysis, the **stage 4 neither group** was used as comparison group, as a reference of patients with less severe disease and, for that reason, with an expected lower risk for worse outcomes.

Patient survival curves were analyzed using Kaplan-Meier method, with comparison between patients’ groups being done by log-rank test, stratified by follow-up time at 12 months. CKD stage 4 groups were explored as predictors of death by extended Cox regression stratified by follow-up time at 12 months, since proportionality was not met. Age, sex, and mCCI, as potential cofounders, were selected as covariates for the extended Cox model.

Incident rate ratio (IRR) of cardiovascular and admission events was calculated by Poisson regression. A two-sided P-value of <0.05 was considered as statistically significant.

Statistical calculations were performed using SPSS, version 24.0 (SPSS Inc., Chicago, IL, USA), and Stata/MP, version 14.1 (Stata Corp, College Station, TX).

## Results

### Baseline Characteristics

The cohort had a mean age of 77 ± 7.4 years old, a median mCCI of 3 (IQR: 1-4), and all patients were Caucasian. In 59% of the patients, referral was made by the primary care practitioner, and as for the rest, referral was made from other specialties or after admission in our inward department. The median eGFR defined by sCr was 39 (28 - 50) mL/min/1.73 m2 and 33 (25 - 44) mL/min/1.73 m2 when defined by sCys. Participants were followed-up during a median time of 29 (IQR: 26 - 33) months.

Comparison of the baseline characteristics of the four groups is shown in Table 1. After excluding the **CKD stage 4 neither** group, there were no significant differences in age, gender, or comorbidities defined by either sCr or sCys between the groups. Unsurprisingly, patients in the **CKD stage 4 neither** group were younger, majority was male, had less heart failure, arrhythmia, and cerebrovascular disease, and consequently, presented a lower mCCI score.

**Table 1:** Comparison of baseline characteristics of the four CKD stage 4 groups

Baseline Characteristics	1.CKD stage 4 Neither n=158 (45%)	2.CKD stage 4 sCr only n=21 (6%)	3.CKD stage 4 sCys only n=62 (18%)	4.CKD stage 4 combined n=107 (31%)	P excluding group 1
**Age, mean ± SD**	75.0±6.7	76.4±6.6	78.2±7.8	79.4±7.5	0.197
**Female (%)**	38	48	56	58	0.683
**mCCI (IQR)**	2 (1-4)	3 (2-4)	3 (1-5)	3 (2-5)	0.483
**EPI_sCr mL/min, median (IQR)**	50	27	39	25	**<0.001**
	(43-67)	(25-29)	(35-45)	(21-28)	
**EPI_sCys mL/min, median (IQR)**	45	35	27	23	**<0.001**
	(39-62)	(33-40)	(24-29)	(19-27)	

CKD: chronic kidney disease; mCCI: modified Charlson Comorbidity Index; sCr: serum creatinine; sCys: serum cystatin C; SD: standard deviation; IQR: interquartile range.

### Primary Outcomes

By the end of the follow-up period, 54 patients had died and only 4 initiated dialysis. No difference between the groups was observed considering patient death (overall or by cause) and ESRD at the end of the follow-up (Table 2). Cardiovascular and infection were the main causes of death.

**Table 2:** Primary outcomes compared between the CKD stage 4 groups

Primary Outcomes	1.CKD stage 4 Neither n=158 (45%)	2.CKD stage 4 sCr only n=21 (6%)	3.CKD stage 4 sCys only n=62 (18%)	4.CKD stage 4 combined n=107 (31%)	P excluding group 1
**Patient death, n (%)**	15 (9)	4 (15)	11 (18)	24 (22)	0.810
**Causes of death, n (total n=54)**					0.217
**Cardiovascular**	4	4	5	12	
**Infection**	5	0	3	10	
**Neoplasia**	5	0	1	2	
** **	1	0	2	0	
**Others/unknown**	(39-62)	(33-40)	(24-29)	(19-27)	
**Dialysis initiation, n**	0	1	1	2	0.663

CKD: chronic kidney disease; sCr: serum creatinine; sCys: serum cystatin C.

As proportionality was not met, survival analysis was stratified by follow-up time at 12 months. 

In the first year, survival curves of the **CKD stage 4 combined** and **sCys only** groups were significantly lower (P=0.028) when compared to the **CKD stage 4 neither** and **sCr only** groups. However, this difference was not found after 12 months (P=0.148).

Similarly, **CKD stage 4 combined** and **sCys only** groups were better predictors of early (<12 months) death in both unadjusted and adjusted extended Cox models (Table 3). Importantly, only **CKD stage 4 sCys only** group was an independent predictor of early mortality in the adjusted model. No differences were detected for the risk of late mortality between the groups in any of the models analyzed. 

**Table 3:** Extended Cox regression exploring predictors of death, considering CKD stage 4 groups at two time-periods

			Unadjusted		Adjusted	
	n per group	n events	HR (95% CI)	P	HR (95% CI)	P
CKD stage 4						
**[0-12 months]**	158	5	Ref.	Ref.	Ref.	Ref.
Neither	21	0	- (no events)	-	- (no events)	-
sCr only	52	7	3.7 (1.2-11.7)	**0.025**	3.5 (1.1-11)	**0.033**
sCys only	107	11	3.4 (1.2-9.8)	**0.024**	2.252 (0.80-6.6)	0.138
Combined						
CKD stage 4						
**[12-36 months]**	153	10	Ref.	Ref.	Ref.	Ref.
Neither	21	4	2,7 (0,8-8,5)	0,099	2,2 (0,7-7,1)	0,188
sCr only	55	4	1,1 (0,4-3,6)	0,839	1,1 (0,3-3,4)	0,911
sCys only	96	13	2,2 (1-5)	0,060	1,5 (0,7-3,6)	0,330
Combined						

Adjusted to: Age, Sex, mCCI

CKD: Chronic kidney disease; mCCI: Modified Charlson Comorbidity Index; Ref. : Reference; sCr: Serum Creatinine; sCys: serum cystatin C.

### Secondary Outcomes

After excluding group 1 (**CKD stage 4 neither**), in which the occurrence of CV events, all-cause admissions, admissions due to AKI, or infectious events was lower, there were no differences between the rest of the groups for CV events, all-cause admissions, and admissions due to AKI. Differently, infectious events seemed to occur in a higher percentage in **CKD stage 4 combined** group (Table 4).

**Table 4:** Occurrence (at least 1) of CV events, all-cause admissions, admissions due to AKI, and infectious events in the CKD stage 4 groups

Secondary Outcomes	1.CKD stage 4 Neither n=158 (45%)	2.CKD stage 4 sCr only n=21 (6%)	3.CKD stage 4 sCys only n=62 (18%)	4.CKD stage 4 combined n=107 (31%)	P excluding group 1
**With CV events, n (%)**	18 (11)	4 (19)	16 (26)	22 (21)	0.717
**With admissions, n (%)**	28 (18)	5 (24)	23 (37)	45 (42)	0.281
**With admissions for AKI, n (%)**	18 (11)	3 (14)	15 (24)	39 (36)	0.067
**With admissions of infectious events, n (%)**	10 (6)	2 (10)	5 (8)	28 (26)	<0.001

AKI: Acute kidney injury; CKD: Chronic kidney disease; CV: Cardiovascular; sCr: Serum Creatinine; sCys: Serum cystatin C.

When calculating incident rate ratio (IRR) for each type of event (Table 5), with an unadjusted model, CV events occurred more often in the **sCys-based only** group and in the **combined** group. However, when using an adjusted model for potential confounders, as age, sex, and mCCI, this difference only remained significant for the **sCys-based only** group. 

**Table 5:** Incident rate ratio of CV events, all-cause admissions, admissions due to AKI, and infectious events in the CKD stage 4 groups

	CV Events	Unadjusted		Adjusted	
	Event rate (100 patients-year)	IRR (95% CI)	**P**	IRR (95% CI)	**P**
**CKD stage 4**	5.3 9.0 12.3 10.3	Ref. 1.7 (0.6-4.6) 2.4 (1.2-4.5) 2.0 (1.1-3.5)	0.227 **0.010** **0.026**	Ref. 1.4 (0.5-3.8) 2.2 (1.1-4.2) 1.5 (0.8-2.8)	0.486 **0.021** 0.214
**Neither**					
**sCr only**					
**sCys only**					
**Combined**					
	**All Admissions events**	**Unadjusted**		**Adjusted**	
	Event rate (100 patients-year)	IRR (95% CI)	**P**	IRR (95% CI)	**P**
**CKD stage 4**	11.5 10.8 29.0 35.5	Ref. 0.9 (0.4-2.2) 2.5 (1.6-3.9) 3.1 (2.1-4.4)	0.885 **<0.001** **<0.001**	Ref. 0.8(0.3-1.8) 2.281 (1.477-3.521) 2.213 (1.507-3.251)	0.543 **<0.001** **<0.001**
**Neither**					
**sCr only**					
**sCys only**					
**Combined**					
	**AKI Events**	**Unadjusted**		**Adjusted**	
	Event rate (100 patients-year)	IRR (95% CI)	**P**	IRR (95% CI)	**P**
**CKD stage 4**	6.0 5.4 17.4 24.0	Ref. 0.9 (0.30-3) 2.9 (1.6-5.1) 3.972 (2.4-6.4)	0.861 **<0.001** **<0.001**	Ref. 0.7 (0.2-2.4) 2.5 (1.4-4.5) 2.6 (1.6-4.4)	0.597 **0.002** **<0.001**
**Neither**					
**sCr only**					
**sCys only**					
**Combined**					
	**Infectious Events**	**Unadjusted**		**Adjusted**	
	Event rate (100 patients-year)	IRR (95% CI)	**P**	IRR (95% CI)	**P**
**CKD stage 4**	3.7 3.6 8.0 14.1	Ref. 1.0 (0.2-4.3) 2.2 (1.0-4.8) 3.8 (2.0-7.2)	0.983 0.054 **<0.001**	Ref. 0.8 (0.2-3.4) 1.8 (0.8-4.1) 2.5 (1.3-4.8)	0.726 0.134 **0.006**
**Neither**					
**sCr only**					
**sCys only**					
**Combined**					

Adjusted to: age, sex, mCCI

AKI: Acute kidney injury; CKD: Chronic kidney disease; CV: Cardiovascular; mCCI: Modified Charlson Comorbidity Index; Ref.: Reference; sCr: Serum Creatinine; sCys: Serum cystatin C.

As for all-cause admissions and admissions due to AKI, there was a higher IRR in the **sCys-based only** group and in the **combined** group for both unadjusted and adjusted models. 

The IRR for infectious events in the **combined** group was two times higher than the IRR in **sCys-based only** group and almost four times higher than the IRR of the **sCr-based** only and **stage 4 neither** group.

## Discussion

In our cohort, people with severe CKD defined by cystatin C had a lower early survival rate when compared to severe CKD defined only by creatinine and patients with CKD from stage 1 to 3. After extended cox regression analysis and adjusting for age, sex, and mCCI, just the cystatin C_only group remained as a predictor of early death. This tendency was also verified when analyzing CV event rate, which was the main cause of death. These results could indicate that serum cystatin C, in comparison to serum creatinine, could represent a better tool for risk stratification for adverse outcomes in old people with severe CKD.

These findings seem to be in accordance with prior reports about the role of cystatin C as a biomarker for CV prediction risk. Shlipak et al. in a meta-analysis study, showed a consistent linear association between the reduction of GFR estimated by CKD-EPI cystatin C-derived equations (cystatin C alone and cystatin C plus creatinine) and increased risk of all-cause mortality and CV mortality, even in cases of mildly reduced kidney function (below 85 mL/min/1.73 m2) 7. The reason why this biomarker is linked to CVD within CKD, according to experimental data, seems to be related to its association in atherosclerotic physiopathology 18.

However, some studies alerted that this association between cystatin C and all-cause plus CV mortality could be due to other cofounding factors, since the populations studied had variable ages and different characteristics as BMI and comorbidities 19. In fact, there was a study that showed that this association was not confirmed in an Australian population of 1165 elderly women aged more than 70 years 20.

In order to exclude cofounding factors that could bias our results, we made an extensive characterization of our baseline population, and among the patients with severe CKD (stage 4) no detectable differences were found concerning cardiovascular risk factors as BMI, blood pressure, diabetes, smoking, dyslipidemia, and concerning organ damage as heart disease, cerebrovascular disease, and peripheral arterial disease.

Considering secondary outcomes, all-cause admissions and AKI admissions, when compared with CKD stage 1 to 3, stage 4 cystatin C_only and stage 4_combined groups had significantly higher IRR of these events. These results concur with the already shown role of cystatin C in predicting all-cause AKI 21.

As for infectious events, the IRR was only significantly higher in the stage 4_combined group. Although there was a significantly higher IRR for the cystatin C_only group in the unadjusted model, this disappeared in the adjusted model.

Hence, in elderly patients with severe non-end stage CKD, sCys-based eGFR seemed to be a better predictor of adverse outcomes than sCr-based eGFR in patients with discordant staging. Patients with stage 4 CKD defined by creatinine alone appeared to behave more alike those with less severe CKD (stage 4_neither), while studied outcomes in patients with stage 4 CKD defined by cystatin C alone were similar to the more severe group defined as CKD stage 4 by both cystatin C and creatinine. 

Other strengths of this study include a large cohort of elderly patients with CKD with an accurate data collection over a 2-year period, an accurate measurement of serum creatinine and cystatin C using standardized assays, and a rigorous statistical analysis. Moreover, the mean age of our patients was significantly higher than in previous studies, giving more evidence to risk prediction in older people, where CKD is particularly prevalent 22.

Nevertheless, our study strengths should be balanced against its limitations. As all participants were Caucasians, generalizations cannot be made. Plus, the absence of information concerning albuminuria represents a weakness of our project, since albuminuria has been reported as an independent predictor of adverse outcomes 23. A larger follow-up time would have strengthened our study, especially for the primary outcome of dialysis start, in order to increase the number of incident cases. Even so, we are aware that elderly patients are more likely to die from any cause than to progress to ESRD, as was observed 24. Differently, for the primary outcome of death, we realize that increasing the follow-up would not change our results, since the differences between the survival rates of the groups vanished after twelve months.

In conclusion, in our cohort, we have demonstrated that the CKD-EPI cystatin C was superior to CKD-EPI creatinine equation in predicting all-cause mortality in the first year, CV events, and all-cause and AKI admissions when used in old patients with severe non-end stage CKD. For that reason, this data cannot be extrapolated to patients with milder stages of CKD. Also, there is a cost-difference between measuring creatinine and cystatin C, therefore clinicians need to understand the usefulness and cost-effectiveness of eGFR based on cystatin C. Nevertheless, its capacity to better predict the likelihood of adverse events and worse outcomes could help in the clinical decision making: to intervene in the group of patients that will benefit the most and to avoid overtreatment in the ones that will not. Further investigations with prospective studies, albuminuria measurement, and cost-effectiveness data are necessary to validate our hypothesis that cystatin C could be a reliable tool to identify patients at a higher risk of adverse outcomes.
